# Four new species of *Cymatodera* Gray from Mexico (Coleoptera, Cleridae, Tillinae)

**DOI:** 10.3897/zookeys.387.6827

**Published:** 2014-03-11

**Authors:** Alan F. Burke, Gregory Zolnerowich

**Affiliations:** 1 Department of Entomology, 123 Waters Hall, Kansas State University, Manhattan, KS 66506, USA

**Keywords:** *Cymatodera*, terminalia, genitalia, antennae, Mexico, checkered beetle

## Abstract

Four new species of *Cymatodera* from Mexico: *Cymatodera bogcioides* **sp. n.**; *Cymatodera pueblae* **sp. n.**; *Cymatodera mitae* **sp. n.**; and *Cymatodera lineata* **sp. n.** are described based on adult male and female specimens. Male genitalia and other characters of taxonomic value are presented.

## Introduction

*Cymatodera*, a group of checkered beetles composed of generalist predators, represents one of the largest genera of North and Central American Cleridae (Barr 1972). The genus has never been revised and much descriptive work at the species level needs to be done. Most described species are known from the southwest United States (Corporaal 1950; Barr 1952), which may be an artifact of uneven collecting. Moreover, the area encompassed by Mexico, especially the central and southern portion of the country, are areas where faunal surveys have been infrequent. Geographically, the terrain of Mexico is very heterogeneous, with a wide array of ecological niches and climates. This cluster of habitats is accompanied by a considerable diversity of organisms, and a number of species of clerids undoubtedly remain to be described from these areas.

Research in progress on *Cymatodera* from Mexico, which includes localities and data from ~6,000 specimens collected throughout the Americas and revised by the first author, indicates 62 described species present in Mexico. This number is far greater than the 27 species previously provided by Barr (unpublished checklist) and the 15 species listed by Vaurie (1952). Recent work by Rifkind et al. (2010) and Burke (2013) added 8 new species to the Mexican and Central American *Cymatodera* fauna. This paper describes four new species of *Cymatodera* restricted to Mexico.

## Methods

Genitalia extraction and dissection procedures are similar to those outlined by Ekis (1977). Much of the morphological terminology follows the work of Ekis (1977), Rifkind (1996) and Opitz (2010). Male genitalia was considered as a key character for the determination of new species.

Specimens were observed using a Leica MZ APO stereomicroscope. All measurements were made using a stereomicroscope ocular micrometer and the software Leica Application Suite v3.4.0. Images were taken using a Leica DFC 500 digital camera and stacked using Auto-Montage v4.00 by Synoptics Ltd.

The following abbreviations are used in the description of the holotypes: TL = Total body length, HW = Maximum head width, HL = Head length, PW = Maximum pronotal width, PL = Pronotal length, EW = Maximum elytral width, EL = Elytral length.

Type material is deposited in the following collections:

CASC California Academy of Science Collection, San Francisco, CA, USA

CNIN Colección Nacional de Insectos, Instituto de Biología, UNAM, DF, México

FSCA Florida State Collection of Arthropods, Gainesville, FL, USA

JEWC James E. Wappes Collection, San Antonio, TX, USA

JNRC Jacques Rifkind Collection, Valley Village, CA, USA

RFMC Roy F. Morris Collection, Lakeland, FL, USA

WFBM William F. Barr Museum, University of Idaho, Moscow, ID, USA

## Descriptions

### Cymatodera bogcioides

Burke sp. n.

http://zoobank.org/34599650-FB03-43C7-B870-9F199A1AF300

http://species-id.net/wiki/Cymatodera_bogcioides

Figs 1 , 6 , 11 , 16 , 17 , 26 , 30 , 31

#### Type material.

**Holotype:** male, Mexico, Jalisco, Careyes, Hotel Costa Careyes, 7-VII-1991, tropical deciduous forest, at light, J. Rifkind and P. Gum, printed red label, holotype deposited in CASC. **Paratypes:** 3 males and 2 females. 1 male and 1 female:  same data as holotype except male collected 4-7-VII-1991 and female collected 6-7-VII-1991 (JNRC); 1 male: Mexico, Jalisco, Estacion Biologica Chamela, 10-20-VII-1985, E. Giesbert (FSCA); 1 male: Mexico, Jalisco, Estacion de Biologia Chamela, UNAM, 14-IX-1993, Black light, Morris, Huether and Wappes, (RFMC); 1 female: Mexico, Jalisco, vic. Chamela UNAM, 19-IX-1993, J. E. Wappes (JEWC).

#### Differential diagnosis.

Males of *Cymatodera bogcioides* are characterized by the presence of a broad, rather deep carina that extends transversely on the first visible ventrite (Fig. 30). *Bogcia oaxacae* Barr, *Cymatodera limatula* Burke, and *Cymatodera obliquefasciata* Schaeffer also have a transversal carina on the first visible ventrite and similar antennae. From these, *Cymatodera bogcioides* is most similar to the sympatric *Bogcia oaxacae* (Fig. 5) and can be distinguished from *Bogcia oaxacae* by the shape of antennomeres 4–11 (Figs 6, 10). *Cymatodera bogcioides* has the antennomeres 4–10 longer than broad and the posterior distal angle of these is somewhat blunt or rounded, the last antennomere is longer than the ninth and tenth antennomeres, and its distal margin is compressed medially (Fig. 6). *Bogcia oaxacae* has antennomeres 4–10 as broad as long and the posterior distal angle sharply pointed, and the last antennomere is about the same length as the tenth antennomere, with its distal margin moderately oblique (Fig. 10). Differences in the protarsal unguis and abdominal segments are also evident for these species. The position of the protarsal claw is very close to the denticle in *Bogcia oaxacae*, but conspicuously separated in *Cymatodera bogcioides*. In addition, the male of *Cymatodera bogcioides* has the posterior margin of the sixth visible ventrite moderately emarginate (Fig. 16), and the posterior margin of the sixth tergite broadly rounded (Fig. 17), while the male of *Bogcia oaxacae* has the posterior margin of the sixth visible ventrite and sixth tergite narrowly truncate (Figs 24–25). The female terminalia of these two species is very similar (Figs 26, 29); as a result, identification of the female of *Cymatodera bogcioides* is only possible in combination with male specimens. Likewise, differences in the male genitalia are also apparent for these species. *Cymatodera bogcioides* has the lateral margin of the tegmen triangular, with the parameres moderately developed (Fig. 11) while *Bogcia oaxacae* has the lateral margins of the tegmen subparallel with the anterior 1/3 strongly oblique, and the parameres are poorly developed (Fig. 15).

**Figures 1–5.**
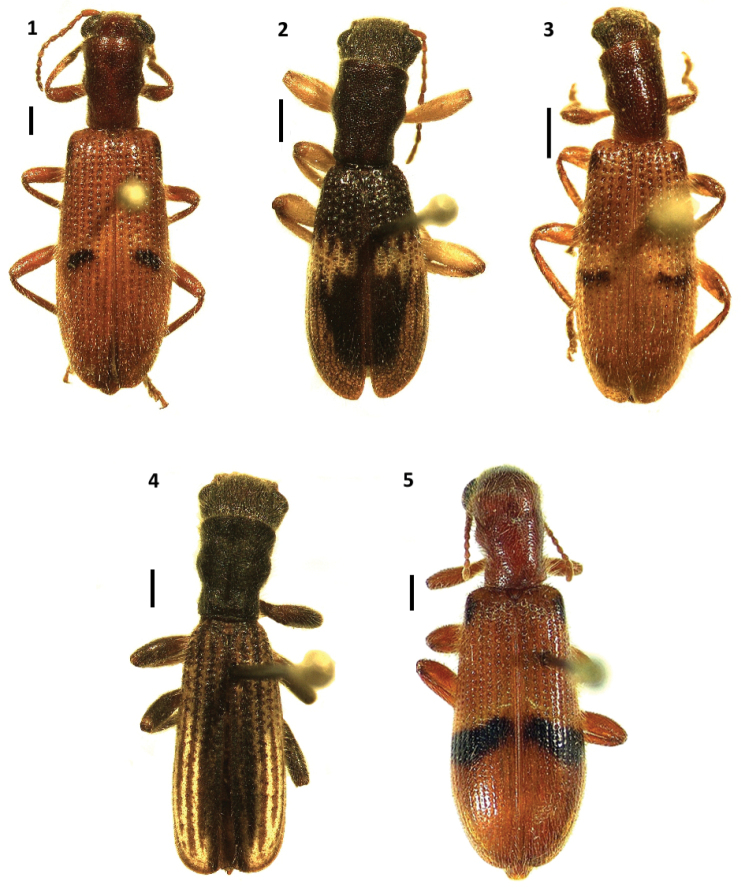
Habitus of: **1** *Cymatodera bogcioides* (holotype male) **2** *Cymatodera pueblae* sp. n. (holotype male) **3** *Cymatodera mitae* sp. n. (holotype male) **4** *Cymatodera lineata* sp. n. (holotype male) **5** *Bogcia oaxacae* (male). Scale bars = 1 mm.

**Figures 6–10.**
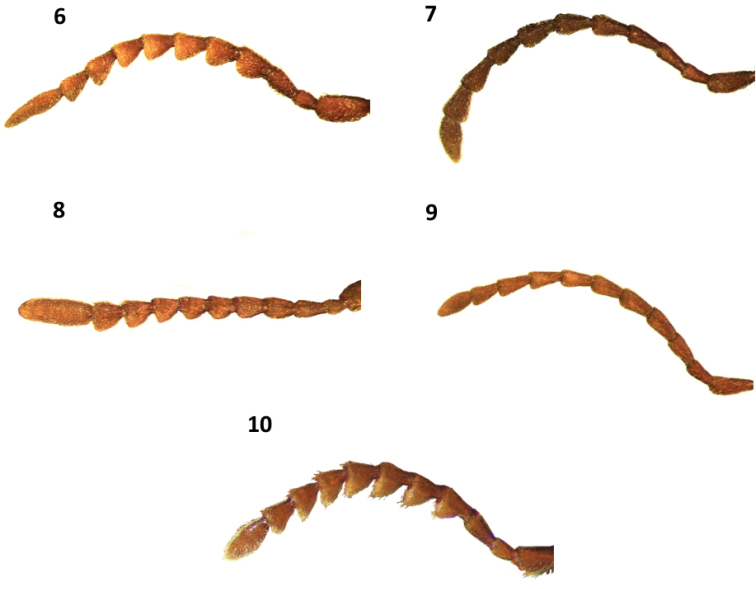
Antennae of: **6** *Cymatodera bogcioides* male **7** *Cymatodera pueblae* (male) **8** *Cymatodera mitae* (male) **9** *Cymatodera lineata* (male) **10** *Bogcia oaxacae* (male).

**Figures 11–15.**
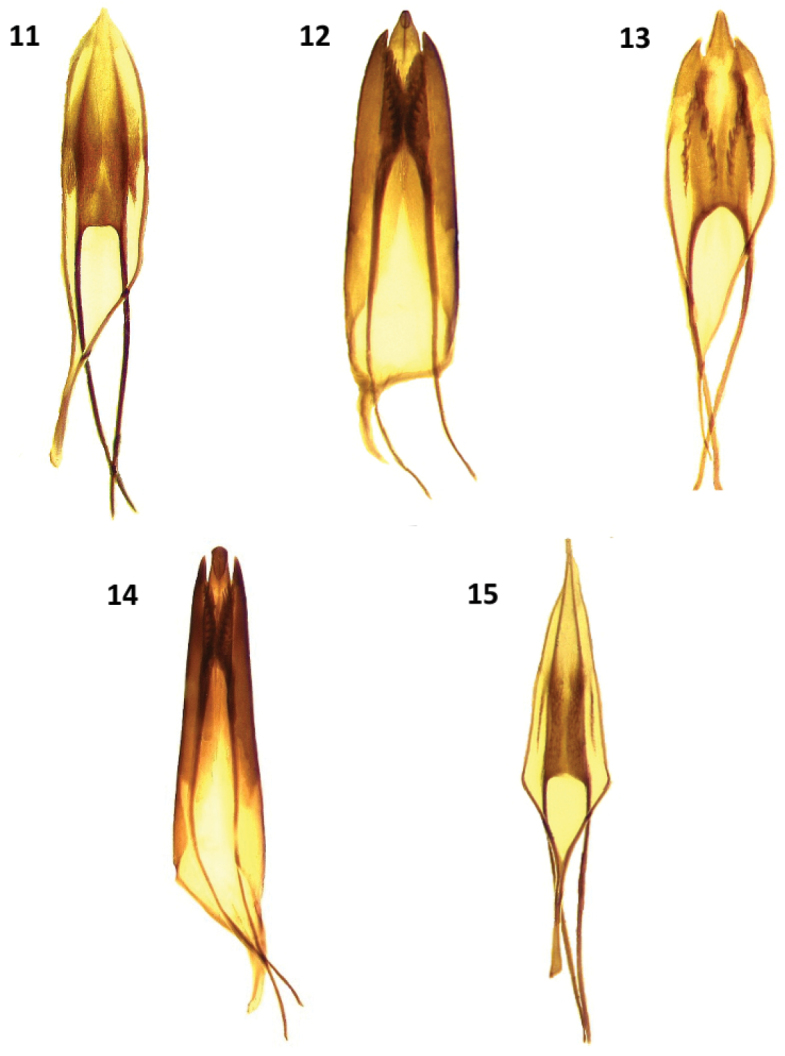
Male genitalia of: **11** *Cymatodera bogcioides* **12** *Cymatodera pueblae* **13** *Cymatodera mitae* **14** *Cymatodera lineata* **15** *Bogcia oaxacae*.

**Figures 16–29.**
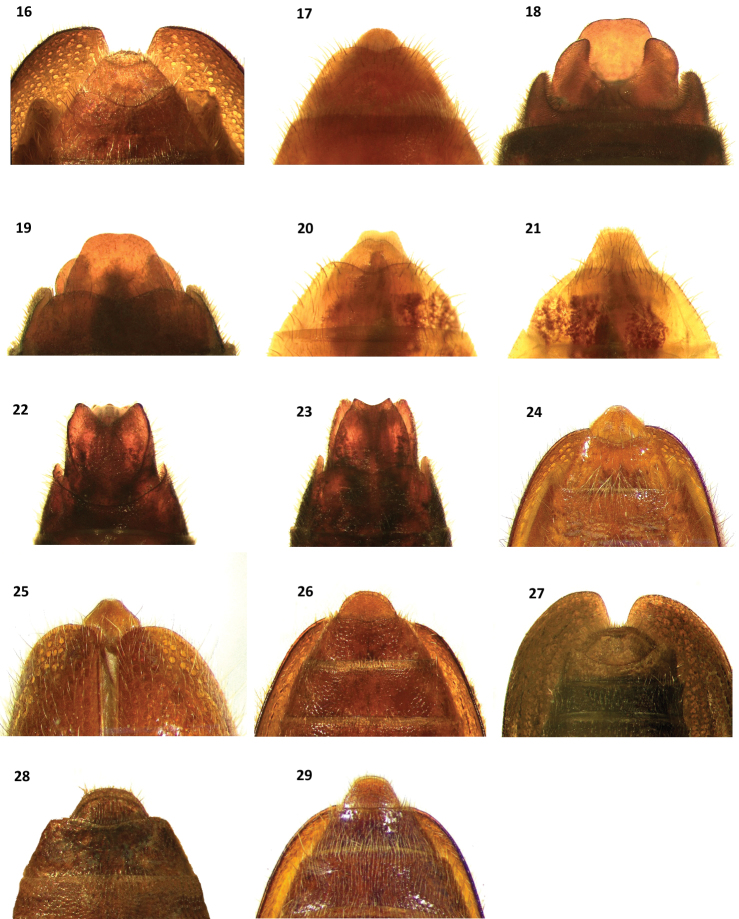
Terminalia of: **16** *Cymatodera bogcioides* (male, ventral view) **17** *Cymatodera bogcioides* (male, dorsal view) **18** *Cymatodera pueblae* (male, ventral view) **19** *Cymatodera pueblae* (male, dorsal view) **20** *Cymatodera mitae* (male, ventral view) **21** *Cymatodera mitae* (male, dorsal view) **22** *Cymatodera lineata* (male, ventral view) **23** *Cymatodera lineata* (male, dorsal view) **24** *Bogcia oaxacae* (male, ventral view) **25** *Bogcia oaxacae* (male, dorsal view) **26** *Cymatodera bogcioides* (female ventral view) **27** *Cymatodera pueblae* (female ventral view) **28** *Cymatodera mitae* (female ventral view) **29** *Bogcia oaxacae* (female, ventral view).

**Figures 30–33.**
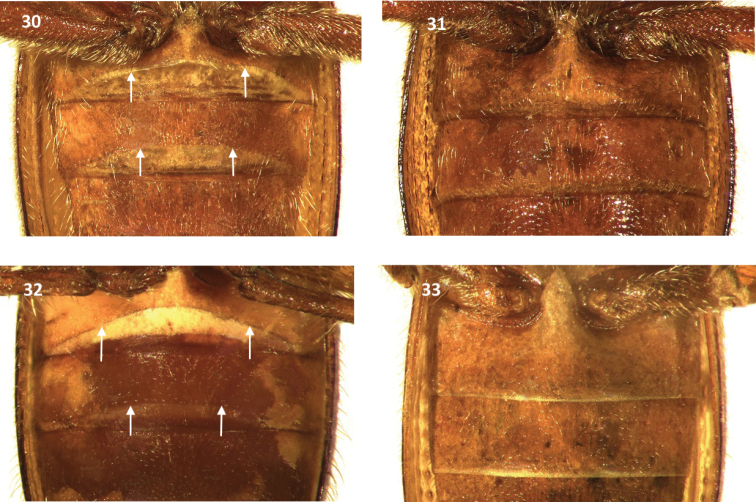
First and second visible ventrites of: **30** *Cymatodera bogcioides* (male) **31** *Cymatodera bogcioides* (female) **32** *Cymatodera mitae* (male) **33** *Cymatodera mitae* (female). Arrows indicate transverse carinae.

#### Description.

Holotype. Medium-sized, rather robust, posterior wings fully developed, TL = 12.75 mm. Color: Head, pronotum, prosternum, mesosternum and metasternum ferruginous, remainder of body uniformly brown. Each elytron with two pairs of dark maculae, the first pair dark brown, located on the humeral angles, the second pair on the median region of the elytral ground, this pair extends from the second to the fifth stria (Fig. 1).

Head: HL = 1.2 mm, HW = 2.2 mm. Measured across eyes wider than pronotum; finely, moderately punctate, vested with semirecumbent setae; surface slightly rugose; frons moderately bi-impressed; eyes large, feebly emarginate in front, rounded, bulging laterally, separated by approximately 1.2 eye-widths. Antennae loosely composed, extending slightly beyond elytral base; third antennomere 2.0× longer than second antennomere, antennomeres 4–10 subequal in length, longer than broad, strongly serrate; blunt at posterolateral portion; last antennomere 2.1× longer than tenth antennomere (Fig. 6).

Thorax: PL = 2.75 mm, PW = 1.85 mm. Pronotum widest at middle; sides constricted subapically, more strongly constricted behind middle; disc flat, not constricted in front of middle; moderately vested with short, semirecumbent setae intermixed with less numerous, semierect setae; surface rather rugose, rugosity becoming more apparent on sides; moderately punctate, punctation somewhat shallow and less numerous on disk; subbasal tumescence feebly indicated. Prosternum smooth, very feebly puncticulate, slightly rugose. Mesosternum moderately, coarsely punctate; scarcely vested with fine, recumbent setae. Metasternum convex; moderately, finely punctate; mesal area with a longitudinal sulcus; covered with fine, recumbent setae.

Legs: Clothed with semirecumbent, semierect, and erect setae of various sizes; femora moderately, shallowly puncticulate, rugulose; tibia moderately, shallowly punctate, rugose; fourth protarsomere with pulvillus medially incised, incision does not extend beyond apical fourth.

Elytra: EL = 7.7 mm, EW = 3.5 mm; broader than pronotum; humeri indicated, rounded; sides subparallel; widest behind middle; disc flattened above; surface shiny, slightly rugose; apices subquadrate; moderately dehiscent; elytral declivity moderately steep; clothed with short, semierect setae intermixed with less numerous, long, semierect and erect setae; sculpture consisting of coarse punctations arranged in regular striae that gradually become smaller and shallower behind posterior 1/4, punctations not reaching elytral apex; interstices at elytral base about 2.5× width of punctation.

Abdomen: Ventrites 1–5 rugose; moderately, finely punctate; clothed with long, fine, recumbent setae. First ventrite convex; subquadrate; posterior margin conspicuously elevated with a transverse carina originating next to posterolateral angles producing a broad, deep, arcuate emargination (Fig. 30). Second visible ventrite somewhat convex; subquadrate; posterior margin slightly elevated with a longitudinal carina producing a moderately broad, rather deep, arcuate emargination. Ventrites 3-4 convex; subquadrate; posterior margin truncate. Fifth visible ventrite convex; lateral margins oblique; posterior margin broadly, deeply emarginate, emargination extending to posterior third of its length; hind angles rounded (Fig. 16). Sixth ventrite subtriangular; surface rugulose; feebly convex; broader than long; lateral margins feebly arcuate, strongly oblique; posterolateral angles rounded; posterior margin broadly, very feebly, shallowly emarginate. Fifth tergite convex; surface rugulose; subquadrate; posterior margin very feebly, narrowly emarginate (Fig. 17). Sixth tergite feebly convex; semicircular; lateral and posterior margins broadly rounded. Sixth tergite extending beyond apical margin of sixth visible ventrite, fully covering sixth ventrite from dorsal view. Aedeagus: 1.95 mm long; ratio of length of paramere to whole tegmen 0.35: 1; tegmen partially covering phallus; parameres moderately developed, pointed at apex; phallobase wide; phallus with copulatory piece acuminate distally; phallic plate devoid of denticles, finely granulate on posterolateral area; phallobasic apodeme rather long, moderately robust distally; endophallic struts slender throughout length (Fig. 11).

Females in the type series differ from males by having the first visible ventrite moderately longer than males, and ventrites 1–2 posteriorly truncate and lacking the moderately elevated transversal carina (Fig. 31). Other abdominal differences in the female are as follows: fifth visible ventrite rugulose; lateral margins oblique; posterior margin shallowly, moderately broadly emarginate. Sixth visible ventrite semicircular; rugulose; feebly convex; lateral and posterior margins broadly rounded (Fig. 26). Fifth tergite rugulose; subtriangular; lateral margins oblique; posterior margin shallowly, moderately broadly and triangularly emarginate. Sixth tergite subtriangular; rugulose; broader than long; surface inconspicuously convex; lateral and posterior margins strongly oblique, producing a rather continuous and semicircular margin. Sixth tergite extending beyond sixth visible ventrite.

#### Variation.

Length of males 12.2–14.9 mm, length of females 12.3–15.2 mm; n = 4. Length to width ratio of head: males average 0.65, females 0.74. Length to width ratio of thorax: males average 1.53, females average 1.49. Length to width ratio of elytra: males average 2.33, females average 2.39. Two males and one female have a slightly more obscure coloration on the elytral ground, these individuals have the humeral maculae completely black, rather than dark brown, as in the holotype.

#### Distribution.

**Figure 34.**
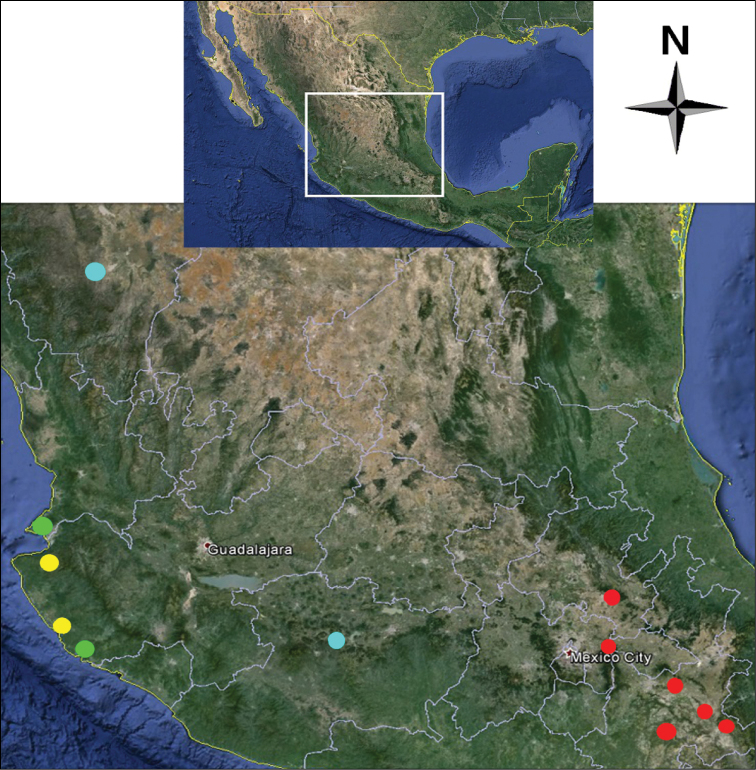
Map of central Mexico showing geographic position of collecting localities for: *Cymatodera bogcioides* (yellow circles); *Cymatodera pueblae* (red circles); *Cymatodera mitae* (green circles); and *Cymatodera lineata* (light blue circles).

The type series was collected in two localities close to each other in the western portion of the state of Jalisco, Mexico. The first locality is in Costa Careyes, in the Costalegre region, and the second locality is the UNAM Biological Research Station located in the Chamela-Cuitzmala natural reserve (Fig. 34).

#### Etymology.

The specific epithet refers to the resemblance of this species to *Bogcia oaxacae* and *Bogcia disjuncta*.

### Cymatodera pueblae

Burke sp. n.

http://zoobank.org/712E2AE1-2E06-4BD2-AA67-F557F425FEAA

http://species-id.net/wiki/Cymatodera_pueblae

Figs 2 , 7 , 12 , 18 , 19 , 27

#### Type material.

**Holotype:** male, Mexico, Puebla, Highway 18, 6 miles S Esperanza, 14-V-1983, 8100 ft, L. O’Brien and G. B. Marshall, printed red label, holotype deposited in CSCA. **Paratypes:** 2 males and 5 females. 1 male: México, Puebla, San Esteban Necoxcalco, 15-X-1992, C. Mayorga (CNIN); 1 male: Mexico, Mexico, km 41 highway Texcoco – Calpulalpan, 2685 m, 19 30 43 N 98 52 40 W, 3-VII-2001, beating oak, R. L. Westcott (JNRC); 1 female: México, Puebla, km 50 Plan de San Miguel, carretera Huajuapan de León - Oaxaca, 14-VIII-1992, C. Mayorga and E. Barrera (CNIN); 1 female: México, Puebla, Nuevo Vicencio, km 50 Carretera El Seco – Amozoc, 20-V-1995, G. Ortega and E. Barrera (CNIN); 3 females: Mexico, Pachuca, H90, 8000 ft, 6, 9-VII-1937, M. A. Embury (WFBM).

#### Differential diagnosis.

*Cymatodera pueblae* is readily distinguished from similar species and other congeners by its small size, shape, sinuate midelytral fascia (Fig. 2), male terminalia (Figs 18–19), genitalia (Fig. 12), and geographic distribution (Fig. 34). No other species has this combination of characters.

#### Description.

Holotype. Small, rather robust, posterior wings present, brachypterous, TL = 8.67 mm. Color: Head fuscous; pronotum, prosternum, mesosternum, mestasternum, legs and elytra brown; abdomen testaceous mesally, becoming brown toward sides; mouthparts pale testaceous. Each elytron bearing a pale testaceous, median fascia that extends from first stria to epipleuron (Fig. 2).

Head: HL = 1.15 mm, HW = 1.88 mm. Measured across eyes conspicuously wider than pronotum; rugose; frons bi-impressed; strongly, coarsely punctate; clothed with short, semirecumbent setae intermixed with long, semierect and erect setae; eyes moderately large, subsinuate, longer than wide, moderately emarginate in front, feebly bulging laterally, separated by approximately 3.5 eye-widths. Antennae slender; loosely composed; extending slightly beyond posterior margin of elytra; antennomeres 2–3 subequal in length; fourth antennomere slightly longer than third antennomere; antennomeres 4–5 subequal in length; antennomeres 6–10 subequal in length, each slightly shorter than fifth antennomere; antennomeres 5–10 weakly serrate; last antennomere flattened apically, as long as tenth antennomere (Fig. 7).

Thorax: PL = 2.35 mm, PW = 1.67 mm. Pronotum elongate; widest at middle; middle slightly broader than anterior margin; sides constricted subapically; somewhat more constricted behind middle; disc flat; very feebly impressed in front of middle; subbasal tumescence pronounced; surface rugose; moderately coarsely punctate; somewhat vested with short, recumbent setae, intermingled with less numerous, long erect setae. Prosternum wider than long, rugulose, puncticulate. Mesosternum coarsely, deeply punctate. Metasternum convex; reduced in length, rugulose, moderately shallowly punctate.

Legs: Femora clothed with short, recumbent setae interspersed with few erect and semierect setae; tibiae vested with short and long erect and semierect setae; femorae and tibiae transversely, moderately rugose.

Elytra: EL = 5.17 mm, EW = 2.8 mm. Anterior margin arcuately emarginate; narrower than widest portion of pronotum; humeri very feebly indicated; sides subovoid; widest behind middle; disc convex; apex rounded, broadly dehiscent; surface smooth, clothed with intermixed setae of three sizes; sixth tergite exposed dorsally; sculpturing consisting of regular, rather coarse and deep striae that gradually reduce in size after first third of elytral length, striae not reaching posterior 1/4 of elytral length; interstices about 1.5× the width of punctation at elytral base.

Abdomen: Ventrites 1–5 rugulose, shallowly, moderately punctate; each segment with a pair of large, shallow impressions near sides; clothed with short, recumbent setae interspersed with less numerous, long, semi-erect setae. Fifth visible ventrite convex; sides oblique; posterior margin broadly, very deeply emarginate, emargination extends beyond first third of ventrite length (Fig. 18); sixth visible ventrite subquadrate; rugulose; surface strongly concave, excavated; moderately coarsely punctate; lateral margins subparallel; posterior margin broadly, very deeply emarginate, emargination extends to near base of segment; posterolateral angles arcuate, recurved ventrally. Fifth tergite moderately convex; rugulose; lateral margins subparallel; posterior margin broadly, shallowly, triangularly emarginate (Fig. 19). Sixth tergite subtriangular; surface strongly convex, smooth, shiny, shallowly punctate; lateral margin oblique; posterior margin subtruncate, narrowly, very shallowly, triangularly emarginate. Sixth tergite extending beyond sixth visible ventrite, fully covering it from dorsal view. Aedeagus: 1.4 mm long; ratio of length of parameres to whole tegmen 0.68: 1; tegmen robust, fully covering phallus; parameres robust, pointed at apex, lateral margins moderately oblique, procurved, posterior portion feebly curved ventrally; phallobase broad; phallus with copulatory piece somewhat acuminate distally; phallic plate armed with a row of long, posteriorly pointed denticles along dorsal margin; phallobasic apodeme short, robust, dilated distally; endophallic struts slender throughout their length (Fig. 12).

Females differ from males by having the posterior margin of the fifth visible ventrite longitudinally truncate; the sixth visible ventrite is rather convex, rugose and broader than long, the lateral margins are strongly oblique, giving the appearance of a broadly rounded margin (Fig. 27); posterior margin of fifth tergite broadly, very shallowly, triangularly emarginate; sixth tergite subtriangular, rugulose, surface moderately convex, broader than long, lateral and posterior margins strongly oblique, forming a semicircular perimeter.

#### Variation.

Length of males 7.9–9.65 mm, length of females 8.85–10.2 mm; n = 5. Length to width ratio of head: males average 0.71, of females 0.83. Length to width ratio of thorax: males average 1.42, females average 1.6. Length to width ratio of elytra: males average 1.82, females average 1.94. One male and one female are darker than the rest of the type series; the mid-elytral fascia of one female is paler and the legs display a paler, yellowish coloration.

#### Distribution.

The type series of this species was collected at different localities in south-central Mexico, a high-altitude region characterized by various ranges surrounded by semiarid plateaus. Two males and two females were collected in the central and south region of the state of Puebla, Mexico; three females were collected in the vicinity of Pachuca, Hidalgo, Mexico; and one female was collected in km 41, highway Texcoco - Calpulalpan in the State of Mexico, a region that borders the central-western portion of the state of Puebla. The vegetation type in all collecting localities is predominantly a low to mid-altitude mixture of *Quercus*-*Juniperus*-*Cupressus* spp. in association with thorny species (Fig. 34).

#### Etymology.

The specific epithet refers to Puebla, the Mexican state where the holotype was collected.

### Cymatodera mitae

Burke sp. n.

http://zoobank.org/D8B43827-4941-42D9-8DF4-860ECA76A259

http://species-id.net/wiki/Cymatodera_mitae

Figs 3 , 8 , 13 , 20 , 21 , 28 , 32 , 33

#### Type material.

**Holotype:** male, Mexico, Nayarit, 2 km NE Punta de Mita, 26-VII-1990, R. L. Westcott, printed red label, holotype deposited in CSCA. **Paratypes:** 2 males and 3 females. 2 females and 1 male: Mexico, Nayarit, 2 km E Punta de Mita, 30-VII to 2-VIII-1993, C. L. Bellamy (JNRC); 1 female: Mexico, Jalisco, 2 km N Cuitzamala, 10-IX-1988, on dead wood, F. T. Hovore (CNCI). 1 male: Mexico, Nayarit, Playa Piedra Blanca, vicinity of Punta de Mita, 22-VII-1993, tropical deciduous forest, mercury vapor and black light, Rifkind, Bellamy and Reifshneider (JNRC).

#### Differential diagnosis.

This species can be distinguished from congeners by its small size (Fig. 3), antennomeres 4–10 gradually becoming serrate distally, and the conspicuously robust and elongate eleventh antennomere (Fig. 8). The fifth and sixth abdominal segments (Figs 20–21) and genitalia (Fig. 13) of the male also serve to separate this species from remaining *Cymatodera* species. Together with *Cymatodera bogcioides*, *Cymatodera mitae* is part of a group of species characterized by a broad, rather deep carina that extends transversely across the first visible ventrite of males (Figs 30, 32).

#### Description.

Holotype. Small, somewhat robust, posterior wings fully developed, TL = 7.75 mm. Color: head, pronotum, prosternum, mesosternum and metasternum ferruginous-brown, remainder of body uniformly brown. Each elytron with a brown macula located on humeral angle and a pair of irregular, obliquely directed fasciae located on median region of elytral length, the first fascia diffuse, yellowish-testaceous, extending from first stria to epipleuron, the second black, posteriorly adjacent to first fascia, extending from second stria to seventh stria (Fig. 3).

Head: HL = 0.7 mm, HW = 1.35 mm. Measured across eyes wider than pronotum; surface moderately rugose; frons bi-impressed; moderately, coarsely punctate; vested with short, recumbent setae and occasional long, semierect setae behind eyes; eyes rather rounded, moderately large, somewhat longer than wide, feebly emarginate in front, bulging laterally. Antennae not reaching posterior margin of pronotum; first antennomere 0.75× longer than second antennomere, third antennomere about equal in length to first antennomere; fourth antennomere slightly shorter than third antennomere; antennomeres 4–10 subequal in length; antennomeres 2–4 subcylindrical; antennomeres 4–10 gradually becoming serrate toward distal end; last antennomere somewhat robust, cylindrical, about the same length as preceding three antennomeres (Fig. 8).

Thorax: PL = 1.95 mm, PW = 1.25 mm. Pronotum rugose; moderately, finely punctate; less coarsely punctate than head; anterior margin as wide as middle; sides constricted subapically; slightly more constricted behind middle; disc flat, inconspicuously impressed in front of middle; subbasal tumescence moderately produced; surface clothed with moderately long, semierect setae interspersed with long semierect and erect setae. Prosternum smooth, shiny, feebly, shallowly punctate. Mesosternum smooth, shiny; moderately, shallowly punctate. Metasternum with surface rugulose, shiny, moderately, shallowly puncticulate.

Legs: Moderately vested with semirecumbent and semierect setae of three sizes; femora somewhat puncticulate, rugulose; tibia moderately, shallowly punctate, rugulose; fourth pulvillus medially incised, incision not extending beyond apical third.

Elytra: EL = 5.1 mm, EW = 2.35 mm. Broader than pronotum; humeri indicated, rounded; sides subparallel; widest portion behind middle; disc flattened above; surface shiny, rugulose; apices rounded; somewhat dehiscent; elytral declivity gradual; clothed with short, semierect setae intermixed with long, semierect and erect setae, long setae more abundant on anterior 1/4 of elytral ground; sculpture consisting of coarse punctations arranged in regular striae that gradually become smaller and shallower on posterior 1/4 of elytral gound, punctations not reaching elytral apex; interstices at elytral base about 2× the width of punctuation.

Abdomen: Ventrites 1–5 rugulose; strongly, finely punctate; clothed with short, fine, recumbent setae. First ventrite rather convex; subquadrate, posterior margin conspicuously elevated with a transverse carina that initiates on posterolateral angles, producing a broad, deep, arcuate emargination (Fig. 32). Second visible ventrite convex; subquadrate; posterior margin feebly elevated with a longitudinal carina producing a moderately broad, rather deep, arcuate emargination. Fifth visible ventrite moderately convex; subquadrate; surface shiny, moderately, shallowly, finely punctate; lateral margins oblique; posterolateral angles rounded; posterior margin truncate with a median, narrow, shallow emargination (Fig. 20). Sixth visible ventrite small; broader than long; surface feebly convex, shiny; moderately, finely and shallowly punctate; lateral margins strongly convex, hind margin reduced, shallowly, broadly emarginate. Fifth tergite subquadrate, rugulose; lateral margin subparallel, posterior margin broadly, shallowly emarginate (Fig. 21). Sixth tergite subtriangular, broader than long; surface rugulose; lateral margins strongly oblique. Sixth tergite extending beyond apical margin of sixth visible ventrite; base of sixth visible ventrite extending laterally, slightly farther than sixth tergite. Aedeagus: 0.9 mm long; robust; ratio of length of paramere to whole tegmen 0.39: 1; tegmen partially covering phallus; parameres moderately robust; lateral margins obtuse, pointed distally; phallobase wide; phallic plate armed with a long row of large, sharp denticles along dorsal margin; phallobasic apodeme slender, moderately short; endophallic struts slender throughout their length (Fig. 13).

Females in the type series have the first visible ventrite posteriorly truncate and slightly longer than males, and lack the transverse carina (Fig. 33) present in males (Fig. 32). The second visible ventrite also lacks the moderately elevated carina observed in males. Fifth visible ventrite rugulose; lateral margins rather arcuate, feebly oblique; posterior margin truncate and medially narrowly, very shallowly emarginate; sixth visible ventrite rugulose, feebly convex; semicircular; lateral and posterior margins broadly rounded (Fig. 28); fifth tergite rugulose, subtriangular; lateral margins moderately oblique; posterior margin shallowly, broadly, triangularly emarginate. Sixth tergite subtriangular; rugulose; broader than long; surface inconspicuously convex; lateral margins rather arcuate, strongly oblique; posterior margin arcuate, rendering a rather continuous and semicircular posterolateral margin. Sixth tergite extending beyond sixth visible ventrite.

#### Variation.

Length of males 6.9–8.1 mm, length of females 7.15–8.7 mm; n = 6. Length to width ratio of head: males average 0.59, females average 0.66. Length to width ratio of thorax: males average 1.6, females average 1.62. Length to width ratio of elytra: males average 2.18, females average 2.21.Two males and one female show a slightly darker integument on the elytral disc; also, these individuals display the humeral maculae completely black, rather than dark brown, as seen in the holotype. One male in the type series displays a feebly paler coloration on the elytral disc. The black fascia is variably marked among individuals, ranging from strongly marked to rather diffuse.

#### Distribution.

The type series was collected primarily in Punta Mita, at the southwestern tip of the state of Nayarit, Mexico. One female specimen was collected in the Cuitzmala region of Jalisco, about 50 km southeast of Punta Mita, Nayarit (Fig. 34).

#### Etymology.

The specific epithet refers to Punta Mita, Nayarit, Mexico, the locality where the holotype was collected.

### Cymatodera lineata

Burke sp. n.

http://zoobank.org/25C70887-9B54-4386-BE3E-83DC809D5458

http://species-id.net/wiki/Cymatodera_lineata

Figs 4 , 9 , 14 , 22 , 23

#### Type material.

**Holotype:** male, México, Michoacán, km 23 carretera Morelia - Pátzcuaro, 2000 m, 26-V-1988, A. Cadena and L. Cervantes, printed red label, holotype deposited in CNCI. **Paratype:** 1 female: Mexico, Durango, 26 miles W Durango, 13-VII-1974, beating oak, collector unknown (WFBM).

#### Differential diagnosis.

The distinctive dark, longitudinal fasciae on the elytral ground, unique among all *Cymatodera* species, serve to separate *Cymatodera lineata* from those species with a similar metathorax and anterior elytral margin.

#### Description.

Holotype. Moderately long, slender, posterior wings absent, TL = 9.9 mm. Color: Head, except gular region and pronotum, fuscous; antennae, mouthparts, gular region, prosternum, mesosternum and abdomen, except anterior portion of first visible ventrite, testaceous; legs, metasternum and anterior portion of first visible ventrite brown. Each elytron adorned with five longitudinal, moderately regular, fuscous fasciae; fasciae becoming paler and narrower toward epipleuron; fasciae 2-4 not reaching elytral apex; first and fifth fasciae interconnected at posterior portion of elytra, reaching apex (Fig. 4).

Head: HL = 1.3 mm, HW = 2.25 mm, length to width ratio of holotype 0.58. Measured across eyes conspicuously wider than pronotum; surface rugose; moderately, coarsely punctate; clothed with a set of intermixed moderately long, recumbent and semirecumbent setae; frons inconspicuously bi-impressed; eyes small, subsinuate, taller than wide, feebly bulging laterally, separated by approximately 3.2 eye widths. Antennae extending to base of elytra; third antennomere slightly longer than second antennomere; antennomeres 3–5 subequal in length; sixth antennomere slightly shorter than fifth antennomere; antennomeres 6–10 subequal in length; antennomeres 2–4 subcylindrical; antennomeres 5–10 very feebly serrate; last antennomere subacuminate, about the same length as tenth antennomere (Fig. 9).

Thorax: PL = 2.55 mm, PW = 1.8 mm; length to width ratio of holotype 1.42 mm. Pronotum somewhat elongate; widest at middle, middle slightly wider than anterior margin; sides constricted subapically, more strongly constricted behind middle; disc feebly convex; moderately impressed in front of middle; subbasal tumescences pronounced; surface moderately clothed with short, semirecumbent setae interspersed with erect setae of three lengths; surface feebly rugose, less rugose than head; moderately, shallowly punctate. Prosternum rugulose; surface feebly concave; weakly, shallowly punctate. Mesosternum concave; moderately, coarsely punctate, scarcely clothed with long, erect setae; metasternum conspicuously wider than long; strongly concave; rugulose; moderately, shallowly punctate.

Legs: Femora and tibiae profusely clothed with short, semirecumbent setae interspersed with long, semierect and erect setae; femora and tibiae transversally, moderately rugose; fourth protarsomere with pulvillus medially incised, incision not extending beyond middle.

Elytra: EL = 6.05 mm, EW = 2.8 mm; length to width ratio of holotype 2.16. Base narrower than pronotum; humeri very feebly indicated; sides subparallel; widest at posterior 1/3; disc feebly convex; surface rugose; apices rounded; strongly dehiscent; clothed with erect setae of three sizes; sculpture consisting of coarse punctations arranged in irregular striae that gradually become smaller, shallower and less numerous before apex; interstices at elytral base about 1.5× the width of punctuation.

Abdomen: Ventrites 1–5 rugulose, subquadrate, moderately, shallowly punctate, vested with short, fine, pale, recumbent setae; posterior margin of third and fourth visible ventrite broadly, deeply emarginate. Fifth visible ventrite subquadrate; surface convex; moderately, coarsely punctate; lateral margins subparallel; posterior margin broadly, deeply emarginate; emargination reaching median region; posterolateral angles pointed (Fig. 22). Sixth visible ventrite subquadrate, longer than broad; surface rugose; moderately, coarsely punctate; with a pair of longitudinal carinae that extend from about the base to near posterolateral angles; lateral margins subparallel, posterior margin broadly, deeply triangularly emarginate, emargination extending from near posterolateral angles to basal fourth; posterolateral angles rounded, recurved ventrally. Fifth tergite rugulose, lateral margins subparallel; posterior margin truncate, with a narrow, shallow, triangular emargination on median region (Fig. 23). Sixth tergite subquadrate, broader than long; surface rugulose; lateral margins feebly arcuate, oblique; posterior margin, broadly, shallowly emarginate; posterolateral angles pointed, recurved dorsally. Sixth tergite extending slightly beyond apical margin of sixth visible ventrite, fully covering the latter from dorsal view. Aedeagus: 2.1 mm long; ratio of length of parameres to whole tegmen 0.65:1; tegmen fully covering phallus; parameres robust at base, then gradually becoming slender toward distal end, pointed at apex, lateral margins oblique; phallobase broad; phallus with copulatory piece rounded distally, posteriorly dilated; phallic plate armed with a row of moderately long denticles along dorsal margin; phallobasic apodeme short, robust, dilated distally; endophallic struts slender throughout their length (Fig. 14).

The only female in the type series has the fifth visible ventrite rugulose; surface convex; lateral margins strongly oblique, rather arcuate; posterior margin widely, deeply emarginate. Sixth visible ventrite rugulose; coarsely punctate; moderately convex; lateral margins feebly arcuate, moderately oblique; posterior margin truncate. Fifth tergite rugulose; surface feebly convex; lateral margins oblique, moderately arcuate; posterior margin broadly, shallowly emarginate. Sixth tergite subtriangular; rugulose; coarsely punctate; lateral margins strongly oblique, feeble arcuate; posterior margin narrowly truncate. Sixth tergite extends very slightly beyond sixth visible ventrite.

#### Variation.

The female has a length of 8.2 mm. Length to width ratio of head: 0.52. Length to width ratio of thorax: 1.51. Length to width ratio of elytra: 2.11. The female shows a moderately brownish coloration, somewhat lighter than the male, and the antennomeres 5–10 are filiform, rather than feebly serrate, as observed in the male.

#### Distribution.

The holotype was collected in the state of Michoacán, at the central-western portion of Mexico, on km 23 on the Morelia-Pátzcuaro highway, a region that has suffered extensive logging and was originally covered by a high to mid altitude *Pinus*-*Quercus* association; the female was collected in the state of Durango, 26 km west of the city of Durango, Mexico (Fig. 34).

#### Etymology.

The specific epithet comes from the Latin *linea* (= line) and refers to the longitudinal fasciae on the elytral ground of this species.

#### Comments.

Although it is not usually desirable to describe a new species based on two specimens, this species is strikingly different from all other known *Cymatodera* species. In addition, the urgent need for identifying and cataloging this diverse group of beetles justifies this description, particularly if such species inhabit poorly known or threatened environments (Rifkind 2012).

## Supplementary Material

XML Treatment for Cymatodera bogcioides

XML Treatment for Cymatodera pueblae

XML Treatment for Cymatodera mitae

XML Treatment for Cymatodera lineata
